# The adipokines progranulin and omentin – novel regulators of basic ovarian cell functions

**DOI:** 10.1186/s12958-024-01215-9

**Published:** 2024-04-04

**Authors:** Alexander V Sirotkin, Zuzana Fabová, Barbora Loncová, Maria Bauerová, Abdel Halim Harrath

**Affiliations:** 1 Faculty of Natural Sciences, Constantine the Philosopher University, Tr. A. Hlinku 1, Nitra, 949 74 Slovakia; 2 Department of Zoology, College of Science, King Saud University, Riyadh, Saudi Arabia

**Keywords:** Progranulin, Omentin, Ovary, Hormones, Apoptosis, Proliferation

## Abstract

The present study aimed to examine the effects of progranulin and omentin on basic ovarian cell functions. For this purpose, we investigated the effects of the addition of progranulin and omentin (0, 0.1, 1, or 10 ng/ml) on the viability, proliferation, apoptosis and steroidogenesis of cultured rabbit ovarian granulosa cells. To determine the importance of the interrelationships between granulosa cells and theca cells, we compared the influence of progranulin and omentin on progesterone and estradiol release in cultured granulosa cells and ovarian fragments containing both granulosa cells and theca cells. Cell viability, proliferation, cytoplasmic apoptosis and release of progesterone and estradiol were measured by Cell Counting Kit-8 (CCK-8), BrdU incorporation, cell death detection, and ELISA. Both progranulin and omentin increased granulosa cell viability and proliferation and decreased apoptosis. Progranulin increased progesterone release by granulosa cells but reduced progesterone output by ovarian fragments. Progranulin decreased estradiol release by granulosa cells but increased it in ovarian fragments. Omentin reduced progesterone release in both models. Omentin reduced estradiol release by granulosa cells but promoted this release in ovarian fragments. The present observations are the first to demonstrate that progranulin and omentin can be direct regulators of basic ovarian cell functions. Furthermore, the differences in the effects of these adipokines on steroidogenesis via granulosa and ovarian fragments indicate that these peptides could target both granulosa and theca cells.

## Introduction

Female fecundity is defined by equilibration between ovarian cell proliferation and apoptosis and defines the viability of ovarian cells; growth, atresia and ovulation of ovarian follicles; and oocyte production. These processes are under the control of steroid hormones, whose production is influenced by the cooperation between granulosa cells and theca cells within the ovarian follicle. According to the “two-cell, two gonadotropin” model, in the theca, under the influence of LH, cholesterol is converted to pregnenolone and metabolized through a series of substrates ending in androgen production. Androgens produced by the theca cells transported to the granulosa cells where they are aromatised to estrogens under influence of FSH [1–5].

Ovarian functions and the resulting fecundity are regulated by the metabolism [6–8]. The influence of the metabolic state on reproductive processes is mediated by adipokines, peptide hormones produced by adipose tissue. The role of some adipokines in the control of ovarian cell functions has been well documented [9–11], but the involvement of other adipokines in the control of healthy female reproductive processes has not yet been determined. Examples of such “unknown” adipokines include progranulin and omentin.

The glycoprotein progranulin (granulin) is an epithelial cell-derived growth factor. A number of physiological and medicinal effects of progranulin have been described. This pleiotropic growth factor/adipokine can induce the secretion of inflammatory cytokines; bind to cytokine receptors; and promote cell cycle progression, growth and migration; reparative processes; and resistance to cytotoxic drugs. These effects can promote cancer and neurodegenerative, inflammatory, cardiovascular, rheumatic and metabolic diseases [12–14].

There are indications that progranulin can be involved in the control of ovarian cell functions. The expression of progranulin precursor has been detected in rat reproductive system including hypothalamic nuclei responsible for control of reproductive events, oviduct, ovarian granulosa cells and oocyte, whilst its expression can be regulated by gonadal steroid hormones [15, 16]. Premature ovarian insufficiency was associated with a low progranulin level [17], and a high blood progranulin level was associated with a better response of female ovaries to ovarian stimulation [19]. FSH, a physiological activator of ovarian functions [5], can promote the expression of progranulin in ovarian carcinoma cells [19], while progranulin can promote ovarian cancer cell proliferation [19–21], likely *via* activation of cyclin D1 [21] and an increase in the proportion of cells in the S-phase of mitosis [20]. No effect of progranulin on the viability of cultured cancer cells has been found [19]. To our knowledge, the action, role and targets of progranulin in the control of healthy ovarian cells have not yet been investigated.

The second adipokine, peptide omentin (intelectin), possesses a wide spectrum of physiological and therapeutic features. Its insulin-sensitizing, anti-inflammatory, anti-atherogenic and oxidative stress-decreasing effects have been reported. Based on these effects, its cardiovascular protective, anti-inflammatory, antidiabetic, and anticancer effects have been proposed [13, 22].

Polycystic ovarian syndrome is associated with a reduced human blood level of omentin [23, 24] and with an increased level of omentin in ovarian follicular fluid [25]. These associations might indicate the involvement of omentin in the control of female reproductive processes, but this involvement has not yet been demonstrated.

The present study aimed to examine the effects of progranulin and omentin on basic ovarian cell functions. For this purpose, we investigated the involvement of progranulin and omentin in addition to viability, proliferation, apoptosis and steroidogenesis in cultured rabbit ovarian granulosa cells. To determine the interrelationships between granulosa cells and theca cells, we compared the influence of progranulin and omentin on progesterone and estradiol release in cultured granulosa cells and ovarian fragments.

## Materials and methods

### Preparation, culture, and processing of ovarian granulosa cells

Nineteen ovaries were collected from noncycling New Zealand White rabbits (3 months of age, live weight 2,2 ± 0,1 kg) at the local rabbit farm of the Research Institute of Animal Production, Nitra, Slovakia. The ovaries were individually stored in a thermos with a physiological solution at room temperature and processed within 6 h of slaughter.

In the first series of our experiments, to analyze ovarian cell viability, proliferation, apoptosis, and secretory activity, we gently scraped the ovarian granulosa cells from the inner surface of the washed ovarian follicles (800–1200 μm) via a lancet and isolated them by centrifugation (10 min at 1500 rpm). Follicles with signs of atresia (opaque, hemorrhagic follicles or follicular cysts) have not been used as a source of granulosa cells. Afterward, the cells were resuspended and cultured in sterile Dulbecco’s modified Eagle’s medium (DMEM/F12) and 1:1 medium (BioWhittaker; Lonza, Verviers, Belgium) supplemented with 10% fetal bovine serum (Bio-West, Inc., Logan, UT, USA) and 1% antibiotic-antimycotic solution (Sigma‒Aldrich, St. Louis, MO, USA). The cells were counted using an automated cell counter (Thermo Fisher Scientific, Inc., Waltham, MA, USA), and the concentration was adjusted to the required volume (10^6^ cells/ml medium). The cell suspension was dispensed in 24-well culture plates (NuncTM, Roskilde, Denmark; 1 ml suspension/well) for enzyme-linked immunosorbent assay (ELISA) and in 96-well culture plates for Cell Counting Kit-8 (CCK-8), BrdU (bromodeoxyuridine), and Cell Death Detection Kit (Brand®, Wertheim, Germany; 100 µl/well) assays. The cells were precultured in medium at 37.5 °C in 5% CO_2_ until an 80% confluent monolayer was formed (2 days).

In the second series of experiments, we selected follicular fragments as the experimental model because ovarian steroidogenesis under physiological conditions requires the cooperation of both the granulosa and theca layers of the ovarian follicle (see Introduction) [3, 5]. The tissue near the ovary was removed, and the ovaries were opened with scissors at the site of entry of blood vessels. The connective tissue inside the ovary was gently punctured with a lancet to access the follicles. The follicles were pressed down and separated from the surrounding connective tissues. Afterward, the follicular wall was cut into thin lengthwise fragments (2–4 mm in diameter, weight 15 ± 8 mg). Then, these fragments were washed three times in sterile DMEM/F12 and 1:1 medium (BioWhittaker) and cultured in the same medium supplemented with 10% fetal bovine serum (Bio-West, Inc.) and 1% antibiotic-antimycotic solution (Sigma‒Aldrich) in 24-well culture plates (NuncTM; 1 ml/fragment/well) at 37.5 °C in 5% CO_2_ for 24 h.

After preculture, in both series of experiments, the medium was replaced with fresh medium of the same composition, and granulosa cells and fragments were cultured with and without progranulin (0, 0.1, 1, 10; Active Bioscience GmbH, Hamburg, Germany) or ometin 1 (0, 0.1, 1, 10; Active Bioscience GmbH). The chosen doses of progranulin corresponded the doses of this adipokine, which were efficient in regulation of ovarian cancer cell functions [16, 19–21]. The chosen doses of omentin corresponded the doses of omentin used in the previous in vitro experiments on non-ovarian cells [13, 22] and the level of omentin detected in human ovarian follicular fluid [25]. Immediately before administration to cells, adipokines were dissolved in phosphate-buffered saline (PBS). The control groups were composed of cells not treated with progranulin or omentin.

Immediately after culture, the granulosa cells were processed for the CCK-8 viability test and BrdU and cell death detection assays. The cell concentration was determined by counting on an automated cell counter (Thermo Fisher Scientific, Inc.). The medium conditioned by either granulosa cells or ovarian fragments was stored at -14 °C until ELISA was performed (see below).

### Cell viability assay

Cell viability was measured by using a Cell Counting Kit-8 (CCK-8; Abcam, Cambridge, UK) as recommended by the manufacturer. In brief, 10 µL of CCK-8 solution was added to each well, and the plates were incubated at 37 °C for 24 h. The absorbance (abs) was read at 450 nm by using an ELISA reader (Thermo Fisher Scientific, Inc.). The percentage of proliferative active cells was calculated.

### Proliferation assay

Cell proliferation, based on the measurement of 5-bromo-2′-deoxyuridine (BrdU) incorporation during DNA synthesis, was determined by using a colorimetric cell proliferation ELISA (Roche Diagnostics GmbH, Roche Applied Science, Germany) according to the manufacturer’s instructions. The reaction products were quantified by measuring the absorbance at 450 nm using an ELISA reader (Thermo Fisher Scientific, Inc.).

### Apoptosis assay

Cell apoptosis was evaluated using a Cell Death Detection Kit (Roche Diagnostics GmbH) according to the manufacturer’s instructions. This assay is based on the measurement of the level of cytoplasmic histone-associated DNA fragments as an index of induced apoptotic cell death. The absorbance was read at 405 nm using an ELISA reader (Thermo Fisher Scientific, Inc.).

### Enzyme-linked immunosorbent assay (ELISA)

The concentrations of progesterone and *17β-*estradiol were analyzed in 25 µl aliquots of the incubation medium using an ELISA according to the manufacturer’s instructions (LDN Immunoassays and Services, Nodhorn, Germany). The features of these tests are outlined in Table 1. The accuracy of these ELISAs was tested for culture medium samples through dilution experiments.

**Table 1:** Characteristics of the ELISAs used in experiments

Substance assayed	Specificity of assay (cross-reactivity of antiserum)	Sensitivity of assay (ng/ml)	Coefficient of variation (%)	
			Intra-assay	Inter-assay
**Progesterone**	≤1.1% with 11-desoxycorticosterone, ≤0.35% with pregnenolone, ≤0.30% 17α-OH with progesterone, ≤0.20% with corticosterone, ˂0.10% with estriol, 17β-estradiol, testosterone, cortisone and 11-desoxycortisol, ˂0.02% with DHEA-S and cortisol	0.045	5.4	5.59
***17β*** **-estradiol**	≤9.5% with fulvestrant, ≤4.2% with estrone, ≤3.8% with E2-3-glucuronide, ≤3.6% with E2-3-sulphate, ≤0.4% with estriol, ˂0.1% with androstenedione, 17-hydroxyprogesterone, corticosterone, pregnenolone, E2-17-glucuronide, progesterone, and testosterone	0.0062	6.4	4.5

### Statistical analysis

The data from this study are reported as the means of values that were obtained in three separate experiments performed on separate days with different groups of granulosa cells and fragments, each obtained from at least six ovaries. Each experimental group included four culture wells containing ovarian granulosa cells and fragments. Significant differences between the groups were determined by using ANOVA followed by Tukey’s test, with SigmaPlot 11.0 (Systat Software, GmbH, Erkrath, Germany). Differences were considered to be significant at P values less than 0.05 (*P* < 0.05).

## Results

**Fig. 1:**
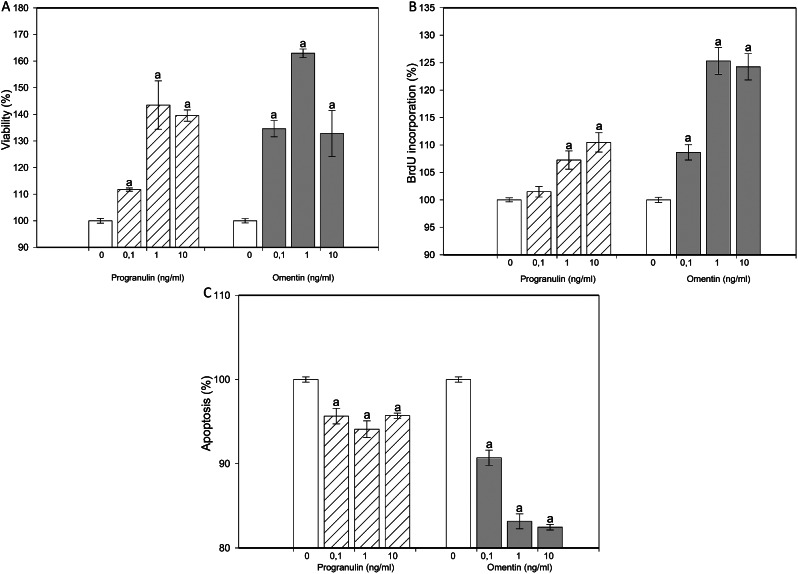
Progranulin, omentin. Effects of the addition of progranulin and omentin at doses of 0 (control), 0.1, 1 and 10 ng/ml on viability (**A**, CCK-8 viability test), proliferation/DNA synthesis (**B**, BrdU test), and cytoplasmic apoptosis/DNA fragmentation (**C**, Cell Death Detection assays) in cultured rabbit ovarian granulosa cells. The results show the effects of the additives: “a” indicates a significant (*P* < 0.05) difference between the cells treated and not treated with these molecules (control, 0 ng/ml). The results are expressed as the mean ± SEM

**Fig. 2:**
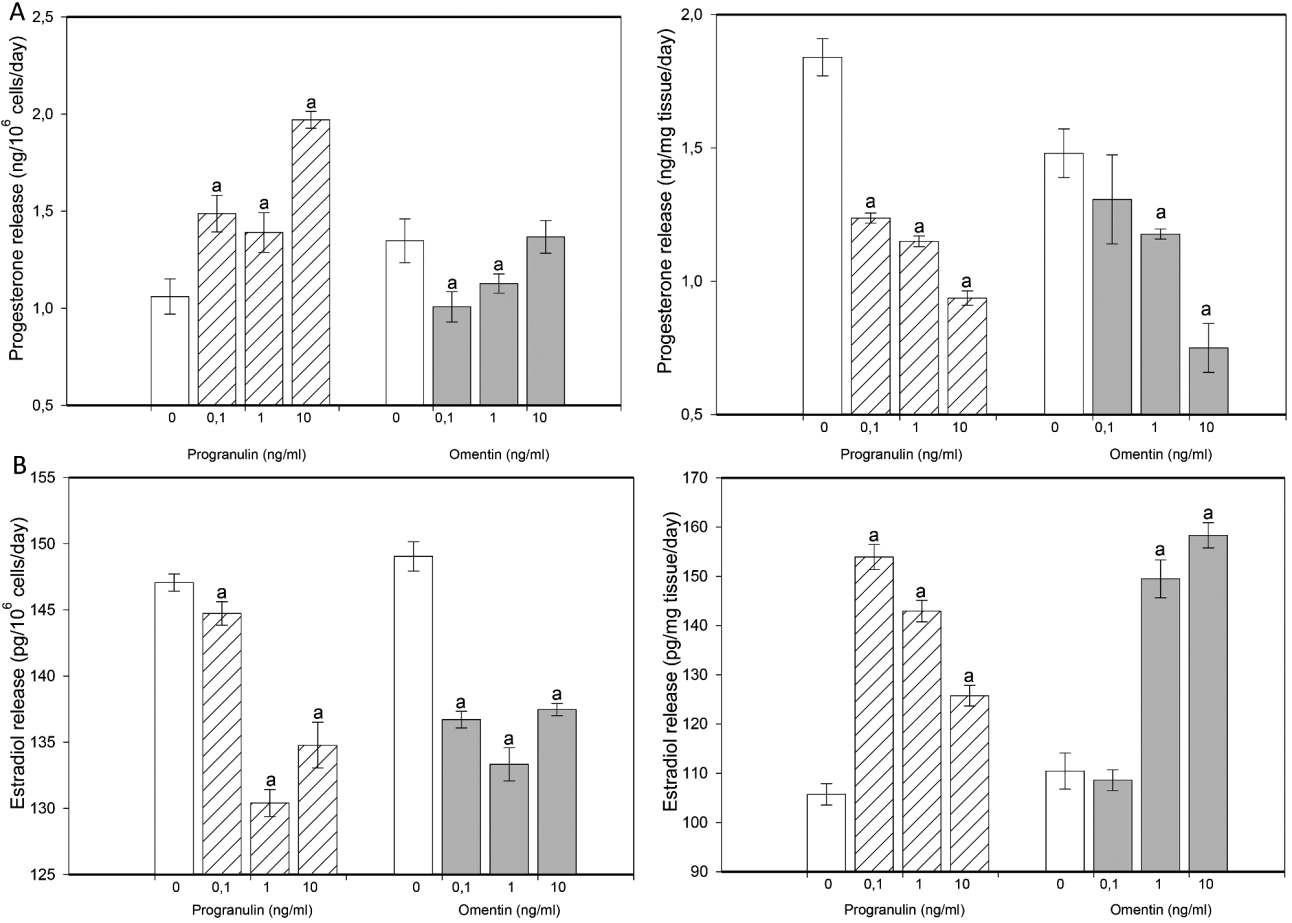
Progesterone Effects of the addition of progranulin and omentin at doses of 0 (control), 0.1, 1 and 10 ng/ml on the release of progesterone (**A**, ELISA) and estradiol (**B**, ELISA) by cultured rabbit ovarian granulosa cells (left) and ovarian fragments (right). The results show the effects of the additives: “a” indicates a significant (*P* < 0.05) difference between the cells treated and not treated with these molecules (control, 0 ng/ml). The results are expressed as the mean ± SEM

When progranulin and omentin were added at all doses, the viability of the cultured granulosa cells increased (Fig. 1A). Progranulin (at doses of 1 and 10 ng/ml) and omentin (at all tested doses) increased granulosa cell proliferation (Fig. 1B). Furthermore, when both molecules were added at all doses, apoptosis decreased (Fig. 1C). Progranulin increased progesterone release by granulosa cells but reduced progesterone output by ovarian fragments at all the tested doses. Omentin reduced progesterone release from both granulosa cells (0.1 and 1 ng/ml) and ovarian fragments (1 and 10 ng/ml) (Fig. 2A). Progranulin decreased estradiol release by granulosa cells but increased it in ovarian fragments at all the tested doses. Omentin reduced estradiol release by granulosa cells (at all doses) but promoted estradiol release in ovarian fragments (at 1 or 10 ng/ml) (Fig. 2B).

## Discussion

The present observations are the first demonstration of direct stimulatory effects of both progranulin and omentin on ovarian cells. In the present study, these peptides increased proliferation and viability and decreased apoptosis in cultured granulosa cells. It is likely that the increase in cell viability induced by progranulin and omentin could be due to an increase in the proliferation: apoptosis rate. These findings are in line with previous reports of the association between some ovarian disorders and production of progranulin [18, 19] and omentin [23–25], which can indicate the involvement of these adipokines in the control of ovarian functions and dysfunctions. Furthermore, the present observations are in line with the previous evidence for the involvement of progranulin in the upregulation of ovarian cancer cell proliferation [19–21] (see Introduction). The present study is the first to demonstrate:

- Direct action of both progranulin and omentin on healthy ovarian cell functions;
- Involvement of both progranulin and omentin in control of animal ovarian functions;
- the involvement of both progranulin and omentin in the regulation of all basic ovarian functions - proliferation, apoptosis, viability and release of hormones;
- that both progranulin and omentin target both granulosa and theca cells of the ovarian follicle.

Understanding the mechanisms/mediators of action of these adipokines on ovarian cells requires further elucidation. There are indications that in nonovarian cells, progranulin [12–14] and omentin [13, 22] can affect oxidative stress and inflammatory processes, which are known regulators of ovarian cell proliferation, apoptosis and viability and result in ovarian folliculogenesis and fecundity [26, 27]. Furthermore, these processes are regulated by steroid hormones [2, 4, 5], which are influenced by both progranulin and omentin and could be additional mediators of their action. The available information enables to hypothesize the role of these adipokines in signaling cascade regulating female reproductive events. Calories intake can promote development of adipose tissue other tissues producing adipokines. Adipokines, via down-regulation of oxidative and inflammatory processes and/or via changes in release of steroid hormones, promote proliferation and viability of ovarian cells, suppress their apoptosis, which in turn can promote of ovarian follicullogenesis and fecundity. This hypothesis requires however validation by in vivo studies.

Steroid hormones are able to affect expression of progranulin [16], while progranulin can affect the release (the present experiments} and the effect [16] of steroid hormones. These facts suggest the existence of the self-regulatory progranulin-steroid hormones axis within the ovary. Progranulin produced by oocyte and granulosa cells, in cooperation with steroid hormones, can be endocrine/paracrine/autocrine regulator of ovarian oogenesis and folicullogenesis.

The similarity in character of influence of progranulin and omentin on ovarian cell viability, proliferation, apoptosis and steroidogenesis (excluding differences in effects of these adipokines on progesterone release by granulosa cells) indicates the similarity in targets and mechanisms of action of progranulin and omentin in the ovary. This hypothesis can be validated by more profound studies of the effects of these adipokines and their combinations.

A comparison of the effects of the studied peptides on steroid hormones released by isolated granulosa cells revealed that the ovarian fragments provided additional evidence for targets of progranulin and omentin in the ovary. It is accepted that ovarian steroidogenesis is a result of cooperation between ovarian follicular granulosa cells and theca cells. Progesterone is produced by both cell layers. Theca cells convert progesterone to androgens, which can be aromatized by granulosa cells to estrogens [1, 3, 5].

In the present study, progranulin increased progesterone release by granulosa cells but reduced progesterone output by ovarian fragments containing both granulosa cells and theca cells. Omentin reduced progesterone release from both granulosa cells and ovarian fragments. The opposite effects of progranulin and omentin on progesterone output by granulosa cells indicate that these two peptides have different mechanisms of action on progesterone release by these cells.

Furthermore, both progranulin and omentin decreased estradiol release by granulosa cells but increased it in ovarian fragments. This similarity in the response of granulosa cells and ovarian fragments to estradiol suggests that granulosa cells are the only source of estrogen production in the ovary. Furthermore, it is suggested that progranulin and omentin have similar targets (granulosa cells), characteristics and probable mechanisms of action on ovarian estrogen release.

In contrast, the opposite effects of the studied peptides on estrogen release by granulosa cells and ovarian fragments containing both granulosa and theca cells demonstrated the importance of theca cells in estrogen production. Furthermore, these findings indicate that both progranulin and omentin regulate ovarian estrogen release, targeting not only granulosa cells but also theca cells. It is possible that in theca cells, these peptides promote the production of androgens, which are aromatized by granulosa cells to estrogen, while a deficit in thecal androgens results in diminished estrogen synthesis by isolated granulosa cells.

However, further studies are needed to validate these hypotheses and discover other mechanisms of action of progranulin and omentin on ovarian cells. Nevertheless, the present observations are the first demonstration of direct effects of progranulin and omentin on basic ovarian cell functions. Furthermore, these findings indicate that these peptides can target both granulosa and theca cells.

The applicability of the obtained data in practice is possible. Previous studies have indicated that progranulin and omentin can be indicators of several reproductive pathologies. The present observations demonstrated that these adipokines can also be physiological regulators of healthy female reproduction. If their role in up-regulation of ovarian functions would be confirmed by in vivo studies, the levels of these adipokines in blood or other biological fluids could be useful as markers and predictive indexes of metabolic and reproductive state. Moreover, administration of these adipokines could be useful for stimulating ovarian functions in farm animals and humans, as well as in treating reproductive insufficiency, infertility, polycystic ovarian syndrome and other animal and human reproductive disorders in reproductive medicine and assisted reproduction.

## Data Availability

No datasets were generated or analysed during the current study.
